# Regulated Expression of Lentivirus-Mediated GDNF in Human Bone Marrow-Derived Mesenchymal Stem Cells and Its Neuroprotection on Dopaminergic Cells *In Vitro*

**DOI:** 10.1371/journal.pone.0064389

**Published:** 2013-05-22

**Authors:** Wei-Hua Yang, Chun Yang, Yue-Qiang Xue, Tao Lu, Jakob Reiser, Li-Ru Zhao, Wei-Ming Duan

**Affiliations:** 1 Department of Anatomy, Capital Medical University, Beijing, China; 2 Department of Cellular Biology and Anatomy, Louisiana State University Health Sciences Center, Shreveport, Louisiana, United States of America; 3 Gene Therapy Program, Louisiana State University Health Sciences Center, New Orleans, Louisiana, United States of America; 4 Department of Neurosurgery, Upstate Medical University, Syracuse, New York, United States of America; University of South Florida, United States of America

## Abstract

Gene regulation remains one of the major challenges for gene therapy in clinical trials. In the present study, we first generated a binary tetracycline-on (Tet-On) system based on two lentivirus vectors, one expressing both human glial cell line-derived neurotrophic factor (hGDNF) and humanized recombinant green fluorescent protein (hrGFP) genes under second-generation tetracycline response element (TRE), and the other expressing the advanced reverse tetracycline-controlled transactivator - rtTA2S-M2 under a human minimal cytomegalovirus immediate early (CMV-IE) promoter. This system allows simultaneous expression of hGDNF and hrGFP genes in the presence of doxycycline (Dox). Human bone marrow-derived mesenchymal stem cells (hMSCs) were transduced with the binary Tet-On lentivirus vectors and characterized *in vitro* in the presence (On) or absence (Off) of Dox. The expression of hGDNF and hrGFP transgenes in transduced hMSCs was tightly regulated as determined by flow cytometry (FCM), GDNF enzyme-linked immunosorbent assay (ELISA) and quantitative real time-polymerase chain reaction (qRT-PCR). There was a dose-dependent regulation for hrGFP transgene expression. The levels of hGDNF protein in culture medium were correlated with the mean fluorescence intensity (MFI) units of hrGFP. The levels of transgene background expression were very low in the absence of Dox. The treatment of the conditioned medium from cultures of transduced hMSCs in the presence of Dox protected SH-SY5Y cells against 6-hydroxydopamine (6-OHDA) toxicity as determined by cell viability using 3, [4,5-dimethylthiazol-2-yl]- diphenyltetrazolium bromide (MTT) assay. The treatment of the conditioned medium was also found to improve the survival of dopaminergic (DA) neurons of ventral mesencephalic (VM) tissue in serum-free culture conditions as assessed by cell body area, the number of neurites and dendrite branching points, and proportion of tyrosine hydroxylase (TH)-immunoreactive (IR) cells. Our inducible lentivirus-mediated hGDNF gene delivery system may provide useful tools for basic research on gene therapy for chronic neurological disorders such as Parkinson’s disease (PD).

## Introduction

Parkinson’s disease (PD) is a progressive neurodegenerative disorder resulting in the loss of dopaminergic (DA) neurons and the impairment of motor function. Currently there is no known cure for PD. The mainstay of therapy for PD is still the oral administration of levodopa which is effective at early stage of treatment and eventually becomes ineffective with side effects associated with a long-term administration. There is an imperative need to develop new therapeutic approaches. Alternative therapeutic approaches have been developed in the use of dopamine agonists, neurosurgical treatment and neural transplantation of embryonic tissue. However, all current therapeutic approaches for PD do not arrest or reverse the fundamental neurodegenerative processes of the disease. Substantial evidence shows that neurotrophic factors can prevent nigral DA neurons from dying and improve the cell functions. Among the neurotrophic factors, glial cell line-derived neurotrophic factor (GDNF) has proven to be a potent neurotrophic factor for protection of nigral DA neurons against toxin-induced degeneration *in vitro* and *in vivo* [1]–[4]. As GDNF being a macromolecule with the molecular weight of 32–34 kDa does not pass through the blood-brain barrier, direct infusion of GDNF into the brain is required to achieve a therapeutic purpose. GDNF has been infused into the brain tissue [5] and the ventricles [6] in clinical trials to treat patients with PD. However, protein infusion into the brain is difficult to develop into a long-term therapeutic approach. To obtain sustained delivery, virus vectors-mediated GDNF gene delivery has been developed.

The GDNF gene can be delivered into the brain using *in vivo* and *ex vivo* gene delivery approaches. For the *in vivo* approaches, recombinant adeno, adeno-associated, or lentivirus vectors harboring the GDNF gene are directly injected into the brain and gene delivery effects have been evaluated in intact and lesioned rodents [7]–[10] and primates [11], [12]. It has been demonstrated that neuronal cells in the brain can be efficiently transduced, resulting in long-term transgene expression. However, there are concerns about live virus administration and genetically modifications of host neuronal cells. For the *ex vivo* approaches, live viruses carrying the GDNF gene are used to transduce cells *in vitro* and then transduced cells are transplanted into the brain. For this purpose, neural stem cells [13], [14], an immortalized neural stem cell line [15], primary astrocytes [16], [17], and mesenchymal stem cells [18]–[20] have been used to serve as gene delivery vehicles. Human bone marrow-derived mesenchymal stem cells (hMSCs) are very easily accessible, prepared and cultured. The use of hMSCs can provide unlimited cell sources for gene delivery vehicles. Moreover, the use of adult hMSCs enables to do autologous transplantation and can avoid immune responses. There is accumulating evidence that MSCs can be genetically modified *ex vivo* by standard retroviral techniques and can stably express transgenes robustly *in vivo* following transplantation [18]–[20].

Lentivirus vectors are considered one of the most promising vehicles to efficiently deliver a gene for basic research and gene therapy, due to ability to transduce non-dividing and dividing cells, stable transgene expression, minimal toxicity and immunity, and a large cloning capacity of 9 kb [21]–[23]. Numerous studies have demonstrated that lentivirus vectors can efficiently deliver the GDNF gene into the brain in animal models of PD, and that GDNF transduction protects dopaminergic neurons from neurotoxin-induced cell death [12], [17], [24], [25]. Nevertheless, potential insertional mutagenesis of lentivirus vectors should not be ignored because lentivirus vectors transduce target cells by randomly integrating into the host chromosomes.

Considering the delivery of a therapeutic gene into the brain, one of the major challenges for clinical application remains the necessity of gene regulation in order to achieve the expected therapeutic outcome while avoiding potential limiting side effects related to the over-expression of the transgene. It is generally agreed that the introduction into the brain of a constitutively produced compound that is not normally present in the brain would not be completely harmless. Indeed, several lines of evidence show that continuous overexpression of the GDNF gene at high doses leads to adverse side effects, such as aberrant sprouting in areas outside the striatum and down-regulation of tyrosine hydroxylase (TH) in the preserved DA terminals [26]. In addition, long-term over-expression of GDNF can induce down-regulation of TH in the intact striatum [8], [27]. It is therefore imperative to introduce an inducible vector system. Several systems have been generated to control gene expression by using an exogenous drug either at the transcriptional or translational level. Inducible gene expression systems are potent research tools, and are constantly developed for their use in basic research and clinical application. Among the existing inducible transcriptional gene regulatory systems, the reverse tetracycline (Tet)-controlled transactivator (rtTA)-regulated system is the most widely exploited tool for inducible gene expression. Gossen and Bujard [28] first described this system which was based on a chimeric transcription factor (the tTA transactivator), resulting from the fusion of the bacterial Tet repressor (TetR) with the activating domain of the herpes virus simplex viral protein 16 (*VP-16*). The rtTA-based system requires the presence of Tet to activate gene transcription (Tet-On system). Several improvements of the rtTA have been made to get the better inducibility and reduced background expression [29]–[32]. The Tet-based system has been successfully used to control gene expression in lentivirus vectors *in vitro* [32]–[37]. However, improvements still need to be made for controlling gene expression by inducible lentivirus vectors *in vivo* [13], [38].

In the present study, we attempted to generate a binary Tet-On lentivirus vector system harboring both the human GDNF (hGDNF) and humanized recombinant green fluorescent protein (hrGFP) genes, and transduce hMSCs with the vectors to establish an inducible cellular hGDNF gene delivery system. We characterized the binary Tet-On lentivirus vector system for inducibility of transgene expression in transduced hMSCs. To prove if transgene product, hGDNF is functional, the study was designed to examine whether conditioned medium from the cultures of transduced hMSCs in the presence of doxycycline (Dox) protected SH-SY5Y cells against 6-OHDA-induced toxicity and improved the survival of nigral DA neurons in serum-free culture conditions.

## Results

### Design and production of improved binary Tet-on lentivirus vectors

**Figure 1:**
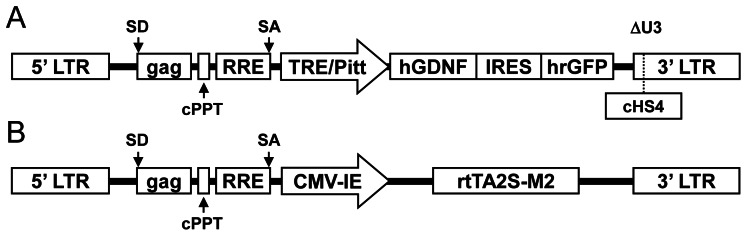
Tet-On inducible lentivirus vector system. (A) Structure of a Tet-regulated transgene vector, pNL-TRE/Pitt-CMV-hGDNF/hrGFP-ΔU3, which carries a TRE/Pitt-hGDNF/hrGFP expression cassette linked to a SV40 poly A signal in reverse orientation with respect to the 5′ LTR. The cHS4 insulator is inserted into the 3′ LTR in the forward (+) orientation. (B) Structure of a transactivator-encoding vector, pNL-CMV-IE-rtTA2S-M2-ΔU3. cHS4, chicken β-globin DNaseI hypersensitive site 4; cPPT, central polypurine tract; gag, 5′ portion of the gag coding region; hGDNF, human glial cell line-derived neurotrophic factor; hrGFP, humanized recombinant green fluorescent protein; IRES, internal ribosome entry site; LTRs, long termimal repeats; RRE, Rev-response element; SA, splice acceptor site; SD, splice donor site; TRE, tetracycline response element; ΔU3, 400-bp deletion of U3 sequences present in the 3′ LTR.

To induce transgene expression in the presence of Dox and reduce background expression in the absence of Dox, Pluta et al. generated a binary Tet-On lentivirus vector system [29]. The improved binary vector system is composed of a self-inactivating (SIN) transgene vector bearing a 400-bp deletion of U3 sequences in the virus 3’ long terminal repeats (LTRs). The transgene vector harbors second-generation tetracycline response elements (TREs) which contain repositioned tetO sequences including TRE/Pitt [39] (Fig. 1A). TRE/Pitt employs 36 bp (3.5 helical turns) between the central bases of two consecutive tetO sequences, and the TATA box of the minimal cytomegalovirus (CMV) promoter was placed 10 bp (1 helical turn) downstream of the first tetO element. The second vector encodes tet-controlled the improved reverse tetracycline-controlled transactivator (rtTA) 2S-M2 [30], in which the herpes simplex virus (HSV) VP16 transactivation domain of the earlier transactivator is replaced with a human E2F4 domain, and it is controlled by the constitutive human minimal CMV-immediate early (IE) promoter (Fig. 1B). In the present study, we modified the transgene vector, and replaced the expression cassette of enhanced GFP (EGFP) with hGDNF/hrGFP. The virus vectors were produced by three-plasmid transient transfection of 293T cells [40], and the titer of the virus vector was around 10^7^ TU/ml.

### Regulated transgene expression with the binary Tet-On lentivirus vector system

**Figure 2:**
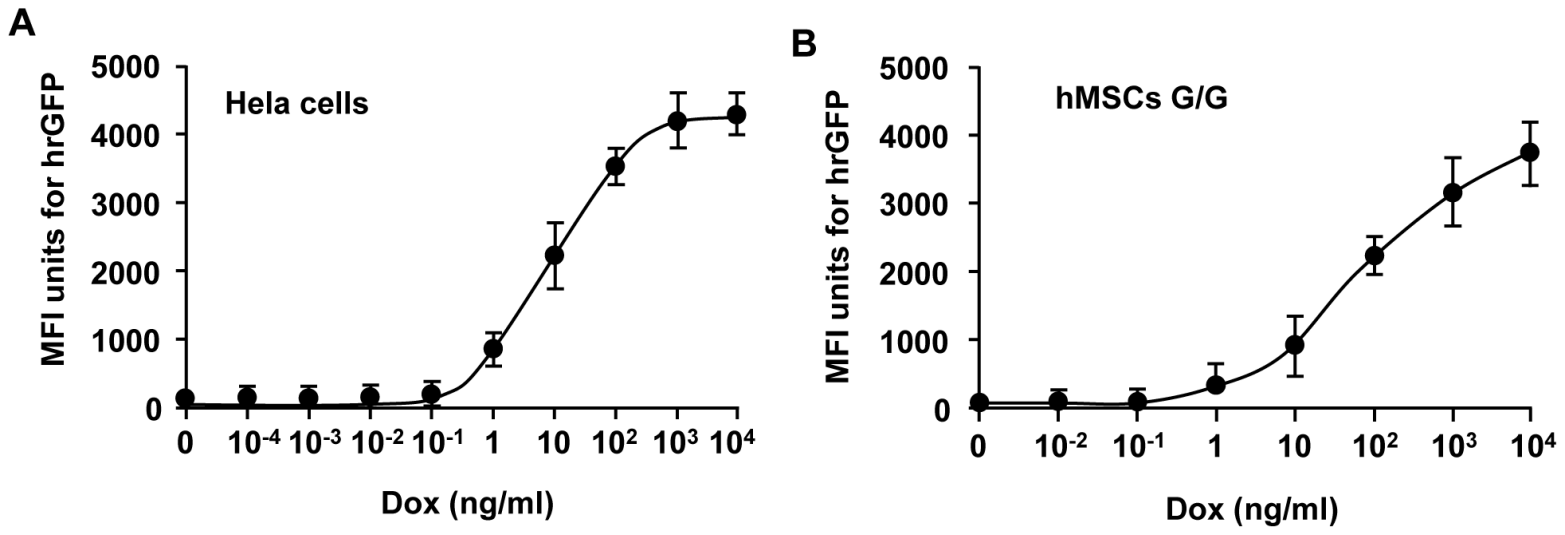
A dose-dependent expression of humanized recombinant green fluorescent protein (hrGFP) gene. HeLa cells and human bone marrow-derived mesenchymal stem cells (hMSCs) were co-transduced with binary Tet-On lentivirus vectors harboring both the human glial cell line-derived neurotrophic factor (hGDNF) and hrGFP genes. The virus vector-containing medium was replaced with the medium in the presence (On) or absence (Off) of doxycycline (Dox) 8 hour after transduction. Serial doses of Dox ranging from 10^−4^ – 10^4^ ng/ml were tested to induce transgene expression. The cells were harvested and mean fluorescence intensity (MFI) units for hrGFP were examined by flow cytometry (FCM) 4 days after Dox treatment. The expression of hrGFP transgene in HeLa cells (A) and hMSCs (B) was regulated in a clear Dox dose-dependent manner.

**Figure 3:**
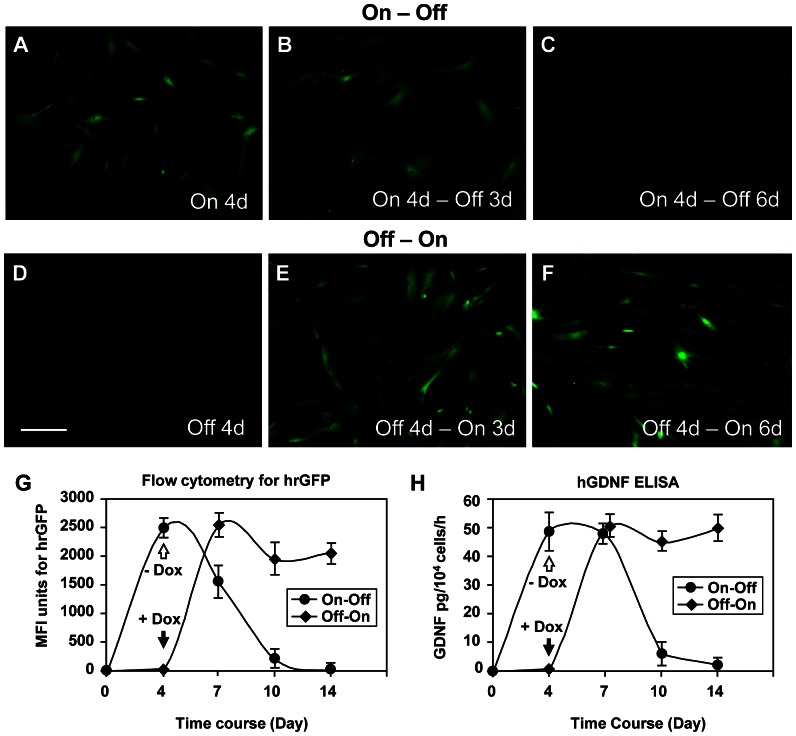
Regulated transgene expression *in vitro*. Photomicrographs (A – C) were prepared from the “On-Off” treatment group in which transduced hMSCs were first incubated in the presence of doxycycline (Dox) (100 ng/ml) for 4 days (A) and then in the absence of Dox for another 3 days (B) and 6 days (C) using an inverted fluorescence microscope. Photomicrographs (D – F) were prepared from the “Off-On” treatment group in which transduced hMSCs were first incubated in the absence of Dox for 4 days (D) and then in the presence of Dox for another 3 days (E) and 6 days (F). Open arrows indicate the time point when Dox is removed from culture medium, and solid arrows depict the time point when Dox is added into culture medium. The scale bar  = 50 µm. (G) Flow cytometry (FCM) analysis showed dynamic changes in the mean fluorescence intensity (MFI) units for hrGFP in the “On-Off” and “Off-On” treatment groups at a desired time points. (H) hGDNF ELISA showed dynamic changes in the levels of hGDNF (pg/10^4^ cells/h) in culture medium in the “On-Off” and “Off-On” treatment groups at a desired time points. Values represent the mean ± standard error of the mean (SEM).

We first examined the inducibility of transgene expression using HeLa cells and hMSCs. The cells were transduced with the binary Tet-On lentivirus vectors, and incubated in the presence of Dox at serial doses ranging from 10^−4^ – 10^4^ ng/ml. Flow cytometry (FCM) analysis showed that mean fluorescence intensity (MFI) units for hrGFP were increased in a Dox dose-dependent manner for both transduced HeLa cells (Fig. 2A) and hMSCs (Fig. 2B). It appeared that hrGFP transgene expression started at a very low dose (0.1 ng/ml) of Dox. We used a dose of 100 ng/ml Dox for the following experiments in the study. We next examined efficiency of regulated transgene expression and the background expression of transgenes in transduced hMSCs in the presence or absence of Dox over time (at 4, 7, 10, and 14 days). No hrGFP^+^ cells were virtually observed in the cultures of transduced hMSCs at the beginning of the experiment (data not shown). For the “On – Off” treated group, numerous hrGFP^+^ cells appeared in the cultures 4 days in the presence (On) of Dox (Fig. 3A). Numerous hrGFP^+^ cells still existed in the cultures 3 days after the removal (Off) of Dox in culture medium (Fig. 3B). However, only a few hrGFP^+^ cells were observed in the cultures 6 days after the removal (Off) of Dox in culture medium (Fig. 3C). For the “Off – On” treated group, the pattern of transgene expression was just contrary to that of the “On – Off” treated group. No hrGFP^+^ cells were observed 4 days *in vitro* in the absence (Off) of Dox (Fig. 3D). Numerous hrGFP^+^ cells were observed in the cultures 3 days after the addition (On) of Dox in culture medium (Fig. 3E). The number of hrGFP^+^ cells remained the same level in the cultures 6 days in the presence (On) of Dox (Fig. 3F). FCM analysis of MFI units for hrGFP confirmed these dynamic changes in hrGFP transgene expression for transduced hMSCs (Fig. 3G). No detectable MFI units for hrGFP were observed in transduced hMSCs at the beginning of the experiment (0±0). For the “On – Off” treated group, MFI units for hrGFP in transduced hMSCs 4 days *in vitro* in the presence (On) of Dox were 2495.56±186.01. After the removal (Off) of Dox at 4 days, MFI units were reduced to 1563.53±321.47 at 7 days (3 days after the removal of Dox), 187.79±73.47 at 10 days (6 days after the removal of Dox), and 4.43±0.37 at 14 days (10 days after the removal of Dox). For the “Off – On” treated group, no MFI units were observed 4 days *in vitro* in the absence (Off) of Dox. After the addition (On) of Dox at 4 days, MFI units were increased to 2508.12±257.68 at 7 days (3 days after the addition of Dox), 1987.16±306.37 at 10 days (6 days after the addition of Dox), and 2106.23±194.43 at 14 days (10 days after the addition of Dox). Interestingly, the levels of hrGFP transgene expression appeared to be reduced in transduced hMSCs at 10 and 14 days in the presence of Dox. Corresponding to the FCM data, GDNF enzyme-linked immunosorbent assay (ELISA) also showed that detectable levels of hGDNF were not observed at the beginning of the experiment, and the levels of hGDNF protein were elevated at 4 days (48.32±7.21 pg/10^4^ cells/h) in the presence (On) of Dox in the “On – Off” group and reduced to a base line after the removal (Off) of Dox (46.68±4.43 at 7 days, 6.71±2.51 at 10 days, and 1.31±0.97 at 14 days) in culture medium (Fig. 3H). For the “Off – On” group, detectable levels of hGDNF were not observed in cultures 4 days (0±0) *in vitro* in the absence (Off) of Dox. After the addition (On) of Dox, the levels of hGDNF were increased to 51.11±5.81 at 7 days (3 days after the addition of Dox) in culture medium, and maintained the same levels at 10 days (45.13±2.06, 6 days after the addition of Dox) and 14 days (50.77±6.41, 10 days after the addition of Dox) *in vitro*. These results suggest Dox can tightly regulate transgene expression in transduced hMSCs, and background expression of transgenes was very low in the absence of Dox.

**Figure 4:**
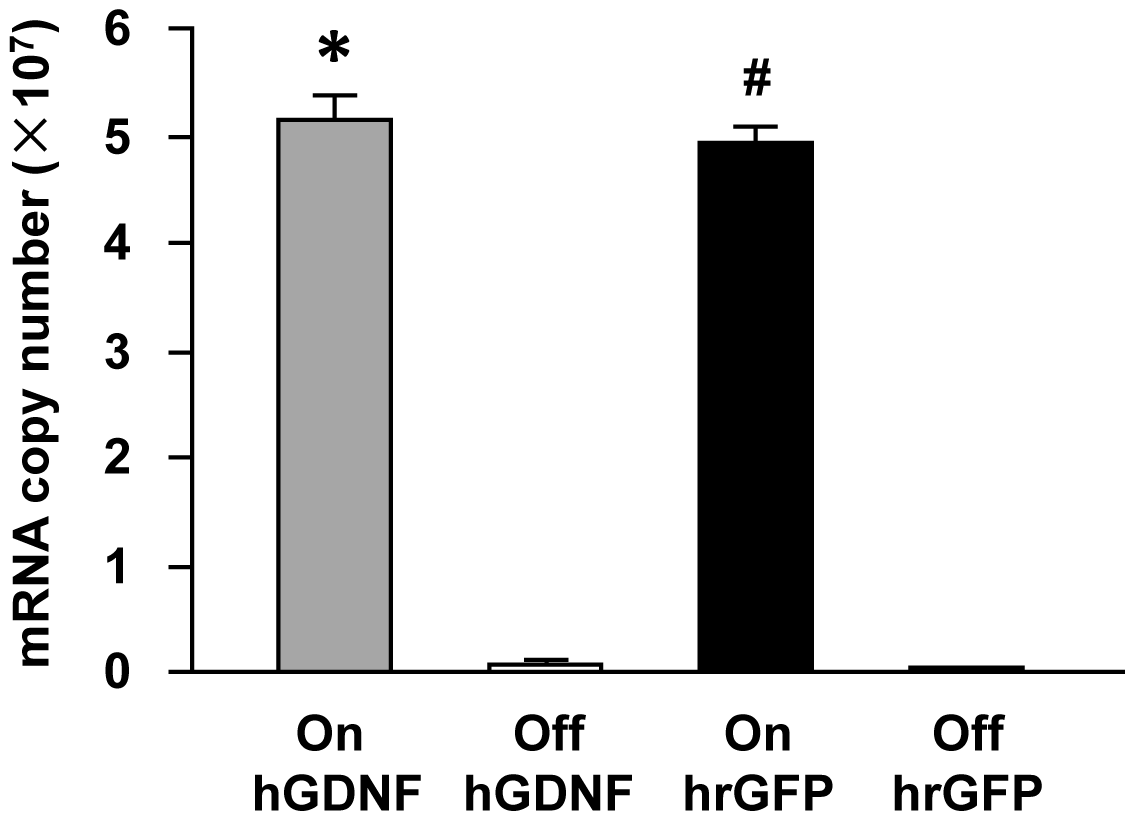
The copy numbers of hGDNF and hrGFP mRNA as determined by quantitative real-time PCR. Transduced hMSCs were incubated in the presence (Grey bar, On hGDNF and black bar, On hrGFP) or absence (Grey bar, Off hGDNF and black bar, Off hrGFP) of Dox (100 ng/ml) for 7 days. Total RNA was extracted from the cells using the Qiagen Rneasy kit. Human β-actin was used as an internal control to normalize the results of hGDNF and hrGFP mRNAs in each sample. Values represent the mean ± standard error of the mean (SEM). A one-factor analysis of variance (ANOVA) followed by Fisher’s post hoc test was applied to make group comparisons. *, # p<0.05 versus Off hGDNF and Off hrGFP groups, respectively.

The above FCM and GDNF ELISA results were confirmed and extended by quantitative real time-polymerase chain reaction (qRT-PCR) using probes specific for hrGFP and hGDNF transcripts. Copy numbers of hGDNF and hrGFP mRNA were examined in the RNA samples isolated from the same cells used for FCM analysis (Fig. 4). The presence of Dox resulted in an average 103-fold increase in copy number of hGDNF mRNA in transduced hMSCs (5.17±0.28)×10^7^ when compared to that in transduced hMSCs in the absence of Dox (0.05±0.02)×10^7^ at 7 days (one-factor analysis of variance, ANOVA, followed by Fisher’s post hoc test, F (3, 20)  = 347.98, * p<0.05). The similar pattern was observed for copy number of hrGFP mRNA. The presence of Dox led to an average 120-fold increase in copy number of hrGFP mRNA in transduced hMSCs (4.78±0.18)×10^7^ when compared to that in the absence of Dox (0.04±0.02)×10^7^ at 7 days (# p<0.05).

### Differentiation of transduced hMSCs

**Figure 5:**
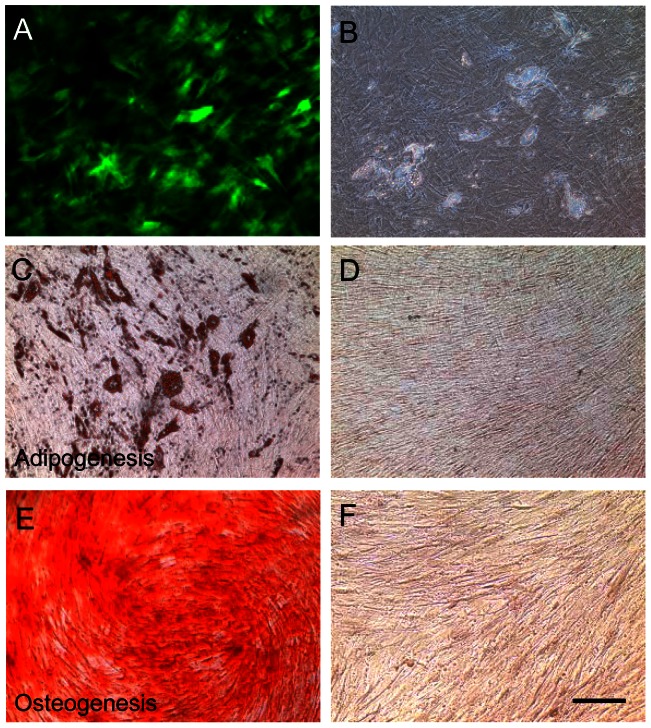
Differentiation of transduced hMSCs. Transduced hMSCs were incubated in either adipogenic or osteogenic differentiation medium in the presence of doxycycline. The cells were fixed with 4% paraformaldehyde and stained with Oil Red O for adipogenic cells (C and D) or Alizarin Red for osteogenic cells (E and F) 3 weeks in vitro. (A) Undifferentiated hMSCs expressed hrGFP. (B) A phase contrast photomicrography of A. (C) Transduced hMSCs differentiated into adipogenic cells versus undifferentiated transduced hMSCs (D). (E) transduced hMSCs differentiated into osteogenic cells versus undifferentiated transduced hMSCs (F). The scale bar  = 50 µm.

It is well known that hMSCs can be induced to differentiate into adipogenic and osteogenic cells due to their stem cell properties. In the presence of Dox, the majority of transduced hMSCs expressed the hrGFP transgene (Fig. 5A and B, a phase contrast image). By incubating in adipogenic or osteogenic differentiation medium, transduced hMSCs were induced to differentiate into adipogenic (Fig. 5C) and osteogenic (Fig. 5E) cells in the presence of Dox 3 weeks after induction. Transduced hMSCs in cultures did not spontaneously differentiate into adipogenic (Fig. 5D) and osteogenic (Fig. 5F) cells. These data suggest that viral transduction does not alter cell properties of hMSCs.

### hMSCs G/G + Dox conditioned medium protects SH-SY5Y cells against 6-OHDA-induced cell death

**Figure 6:**
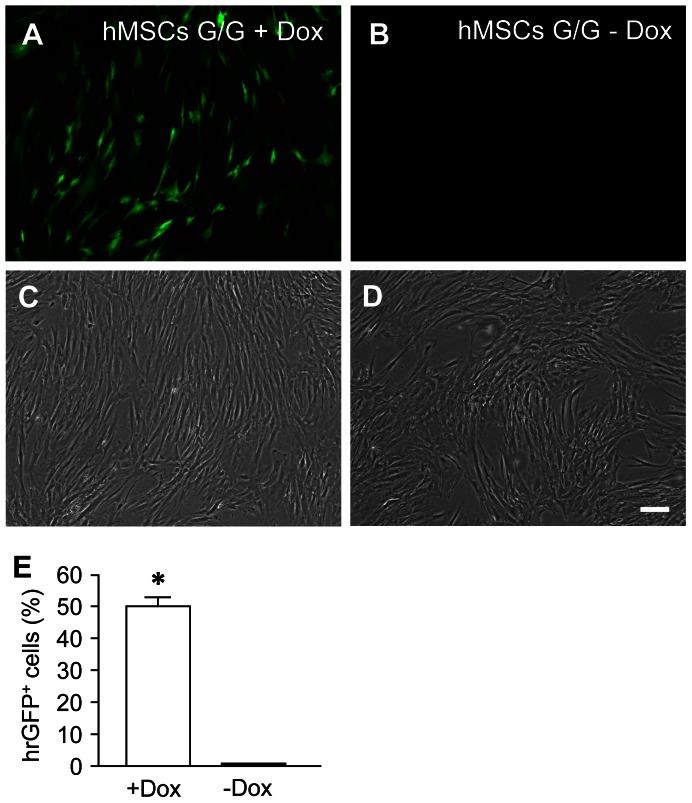
The expression of the hrGFP gene in transduced hMSCs in the presence (On, A, C) or absence (Off, B, D) of Dox (100 ng/ml) at 7 days. Fluorescent (A and B) and phase contrast (C and D) photomicrographs were prepared using an inverted fluorescence microscope. A and C are from the same field and so as B and D. The scale bar  = 100 µm. (E) Flow cytometry (FCM) analysis showed the percentage of hrGFP expressing hMSCs in the presence or absence of Dox. Values represent the mean ± standard error of the mean (SEM). A one-factor analysis of variance (ANOVA) followed by Fisher’s post hoc test was applied to make group comparisons. * p<0.05 versus (−Dox, Off) group.

To examine effects of hGDNF released by transduced hMSCs, conditioned medium was collected from cultures of transduced hMSCs in the presence or absence of Dox at 7 days. At this time point, numerous hMSCs (Fig. 6A) in the total cells (Fig. 6C) in culture expressed hrGFP transgene in the presence of Dox. In contrast, only a few hMSCs (Fig. 6B) in the total cells (Fig. 6D) in the culture expressed hrGFP transgene in the absence of Dox. FCM analysis showed that the percentage of hrGFP-positive cells was much greater in hMSCs in the presence of Dox (50.07±2.76%) than that in hMSCs in the absence of Dox (0.33±0.03%) (Fig. 6E) (one-factor ANOVA followed by Fisher’s post hoc test, F (1, 4)  = 325.78, * p<0.05). Table 1 summarizes the data of hGDNF ELISA. Undiluted hMSCs G/G + Dox conditioned medium contained significant levels of hGDNF (93.45±2.83 ng/ml). In addition, changes in the concentrations of hGDNF in conditioned medium were correlated with serial dilutions of conditioned medium. Detectable levels of hGDNF were not observed in undiluted hMSCs conditioned medium, and only very low levels of hGDNF (0.13±0.00 ng/ml) were detected in undiluted hMSCs G/G − Dox conditioned medium.

**Table 1:** hGDNF ELISA.

Conditioned	hGDNF (ng/ml)			
Medium	Undiluted	1∶80	1∶160	1∶320
hMSCs	0	-	-	-
hMSCs G/G − Dox	0.13±0	-	-	-
hMSCs G/G + Dox	93.45±2.83	1.05±0.03	0.59±0.01	0.27±0.01

**Figure 7:**
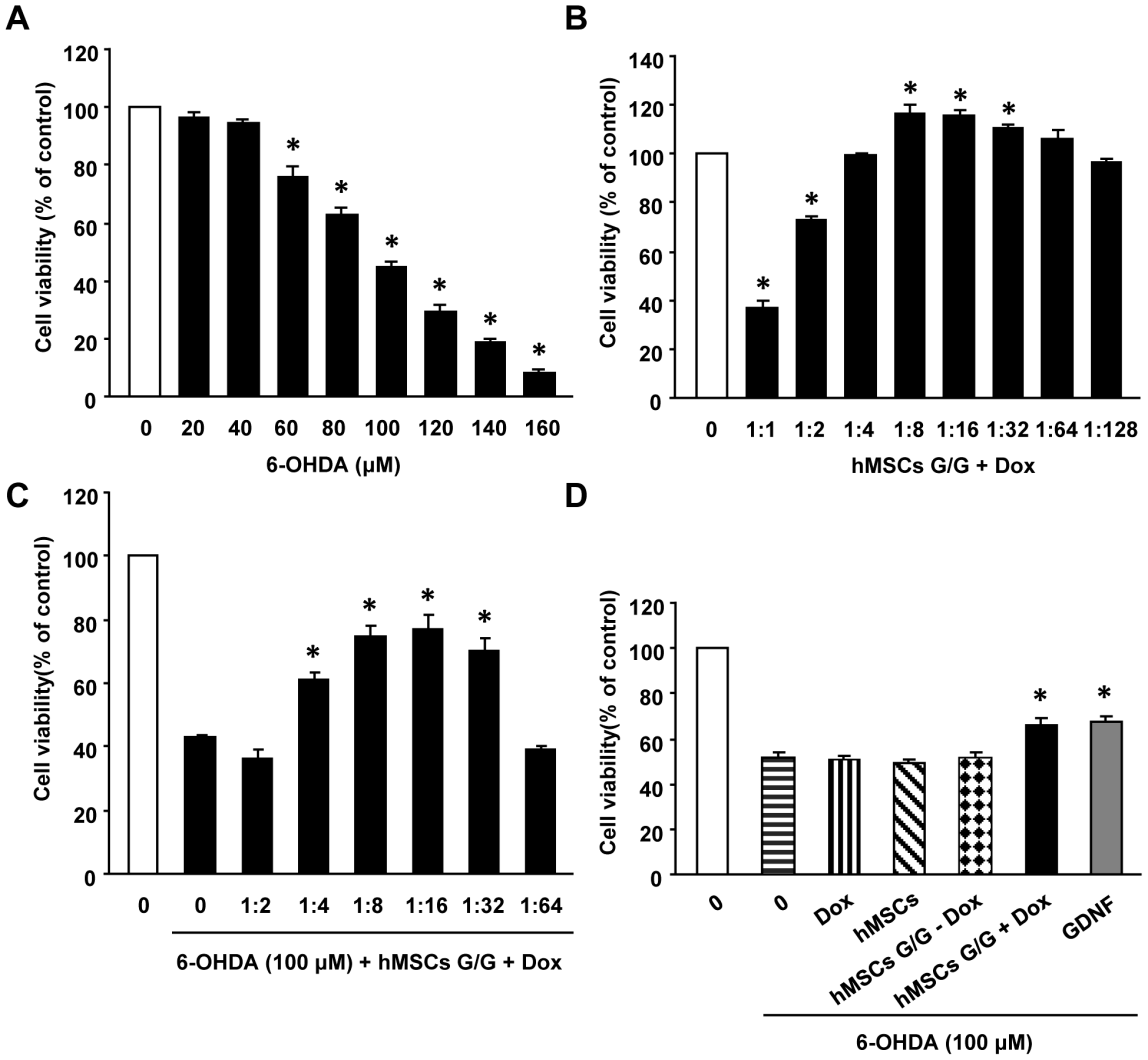
Effects of conditioned medium on SH-SY5Y cells against 6-hydroxydopamine (6-OHDA)-induced toxicity. Cell viability was measured by MTT assay and presented as percentage of the control. (A) Cells were treated with 6-OHDA at serial concentrations ranging from 20 to 160 µM for 16 hours. Cell viability was reduced in a dose-dependent manner. (B) Effects of conditioned medium from transduced hMSC cultures in the presence of Dox (hMSC G/G + Dox) on cell viability of SH-SY5Y cells. (C) Concentration-dependent effects of conditioned medium from hMSC G/G + Dox on 6-OHDA-induced toxicity for SH-SY5Y cells. (D) Effects of various conditioned medium on 6-hydroxydopamine (6-OHDA)-induced toxicity for SH-SY5Y cells. A one-factor analysis of variance (ANOVA) followed by Fisher’s post hoc test was applied to make group comparisons. * p<0.05 versus the control group. Dox, Dox (12.50 ng/ml, equal to the levels of Dox in diluted hMSCs G/G + Dox medium used in the experiment) in DMEM/F12; hMSCs, medium from untransduced hMSCs cultures; hMSCs G/G − Dox, medium from transduced hMSCs cultures in the absence of Dox; hMSCs G/G + Dox, medium from transduced hMSCs cultures in the presence of Dox; GDNF, GDNF (11.68 ng/ml, equal to the levels of GDNF in diluted hMSCs G/G + Dox medium, 1∶8, used in the experiment) in DMEM/F12.

As shown in Fig. 7A, the treatment with 6-OHDA for 16 h resulted in a dose-dependent decrease of SH-SY5Y cell viability (one-factor ANOVA followed by Fisher’s post hoc test, F (8, 36)  = 259.70, * p<0.05). At a concentration of 100 µM, 6-OHDA reduced the cell viability by about 50%, when compared to the vehicle control. We therefore used the concentration of 100 µM of 6-OHDA in the following experiments. To examine the effect of hMSCs G/G + Dox conditioned medium from transduced hMSCs in the presence of Dox alone on SH-SY5Y cell viability, SH-SY5Y cells were treated with the conditioned medium at serial concentrations ranging from 1∶1 to 1∶128 for 16 h (Fig. 7B). Interestingly, the treatment with hMSCs G/G + Dox conditioned medium at concentrations of both 1∶1 (36,61±3.23%) and 1∶2 (72.657±1.61%) reduced cell viability of SH-SY5Y cells when compared to the control (one-factor ANOVA followed by Fisher’s post hoc test, F (8, 36)  = 122.24, * p<0.05). The treatment with hMSCs G/G + Dox conditioned medium at concentrations of 1∶8 (116.47±3.36%), 1∶16 (115.87±2.18%), and 1∶32 (110.75±1.58%) increased cell viability of SH-SY5Y cells (* p<0.05). To examine an optimal concentration of hMSCs G/G + Dox conditioned medium against 6-OHDA-induced cell death of SH-SY5Y, the cells were co-incubated with 6-OHDA (100 µM) and hMSCs G/G + Dox conditioned medium at concentrations ranging from 1∶2 to 1∶64 for 16 h (Fig. 7C). The treatments with hMSCs G/G + Dox conditioned medium at concentrations of 1∶4 (61.32±2.28%), 1∶8 (74.91±2.96%), 1∶16 (77.25±4.38%) and 1∶32 (70.38±3.60%) were found to increase cell viability of SH-SY5Y cells in the presence of 6-OHDA when compared to the cells only exposed to 6-OHDA (42.74±0.90%) (one-factor ANOVA followed by Fisher’s post hoc test, F (7, 31)  = 71.56, * p<0.05). We therefore used hMSCs G/G + Dox conditioned medium at the concentration of 1∶8 in the following experiments. Similar to the treatment with GDNF protein in the culture medium (67.60±2.47%), the co-treatment with hMSCs G/G + Dox conditioned medium increased cell viability of SH-SY5Y in the presence of 6-OHDA (66.07±2.89%) (Fig. 7D) (one-factor ANOVA followed by Fisher’s post hoc test, F (6, 28)  = 66.91, * p<0.05). In contrast, the treatment with Dox, hMSCs and hMSCs G/G − Dox conditioned medium failed to protect SH-SY5Y cells against 6-OHDA-induced cell death. The data suggest that hMSCs G/G + Dox conditioned medium increases cell viability of SH-SY5Y cells, and prevents from 6-OHDA-induced cytotoxicity in SH-SY5Y cells.

### hMSCs G/G + Dox conditioned medium protects dopaminergic cells in cultures

**Figure 8:**
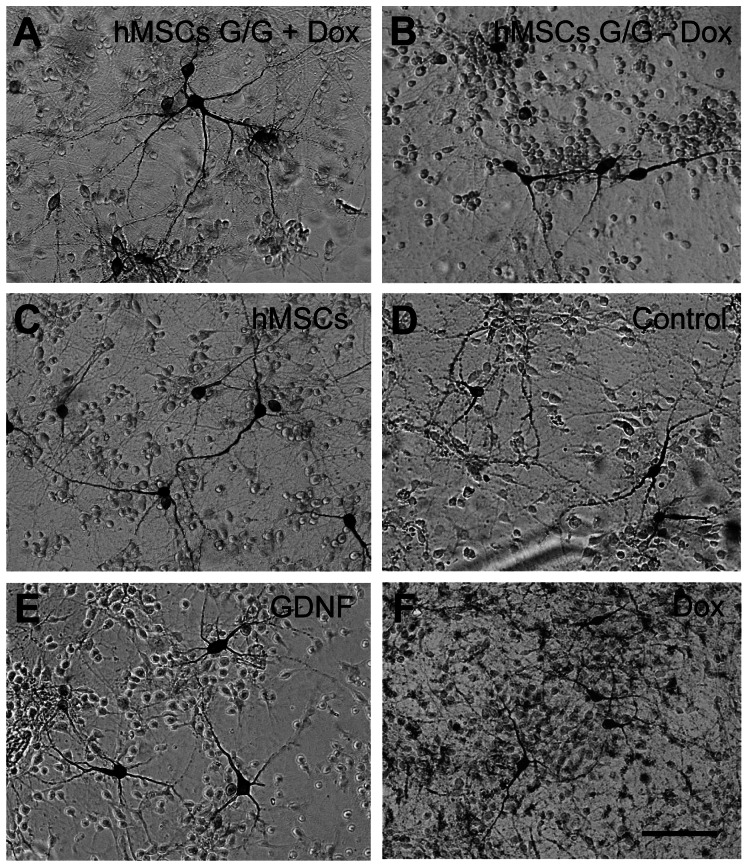
Effects of various conditioned medium on the survival of dopaminergic neurons from ventral mesencephalic (VM) tissue cultures. VM tissue culture was prepared from 14 day-old embryos of Sprague-Dawley rats. After 2 days in culture, medium was switched from DMEM + 10% fetal bovine serum medium to serum-free DMEM/F12 supplemented with N_2_ medium, and some cultures were added conditioned medium. The cultures were immunostained for tyrosine hydroxylase (TH) 7 days *in vitro*. Representative photomicrographs were prepared for (A) hMSCs G/G + Dox, treated with conditioned medium from transduced hMSCs cultures in the presence of Dox; (B) hMSCs G/G – Dox, treated with conditioned medium from transduced hMSCs cultures in the absence of Dox; (C) hMSCs, treated with conditioned medium from no transduced hMSCs culture; (D) Control, treated with conditioned DMEM/F12+N2 medium; (E) GDNF, treated with conditioned medium containing GDNF (11.68 ng/ml, equal to the levels of GDNF in diluted hMSCs G/G + Dox medium, 1∶8, used in the experiment) in DMEM/F12+N2; (F) Dox, treated with conditioned medium containing Dox (12.50 ng/ml, equal to the levels of Dox in diluted hMSCs G/G + Dox medium used in the experiment) in DMEM/F12+N2. The scale bar  = 50 µm.

**Figure 9:**
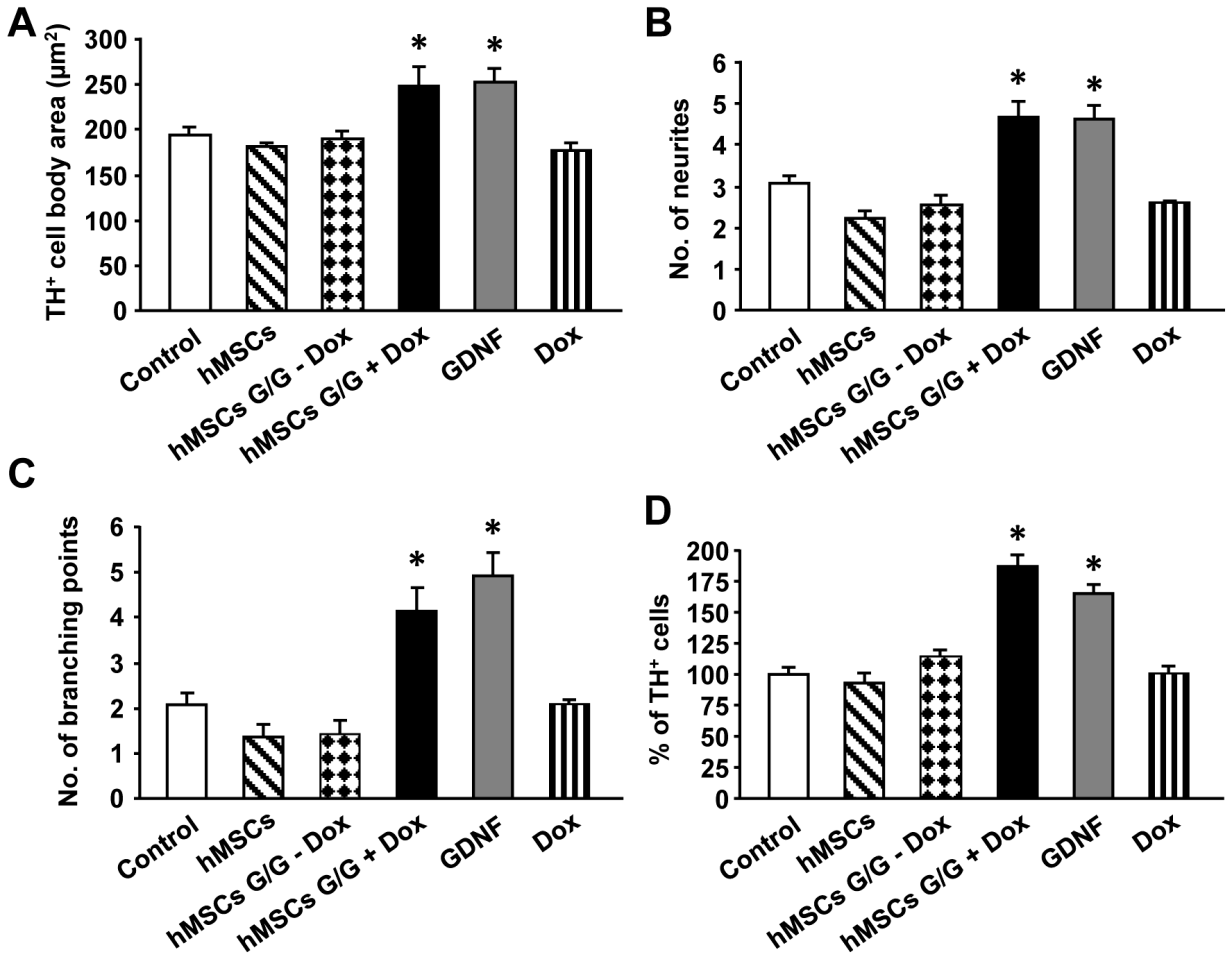
Quantitative data of cell body area (A), number of neurites (B), number of branching points (C), and the proportion of TH^+^ cells (D) are summarized. A one-factor analysis of variance (ANOVA) followed by Fisher’s post hoc test was applied to make group comparisons. * p<0.05 versus the control group. Control, DMEM/F12+N2 medium; hMSCs, medium from no virally-transduced hMSCs cultures; hMSCs G/G – Dox, medium from transduced hMSCs cultures in the absence of Dox; hMSCs G/G + Dox, medium from transduced hMSCs cultures in the presence of Dox; GDNF, GDNF (11.68 ng/ml, equal to the levels of GDNF in diluted hMSCs G/G + Dox medium, 1∶8, used in the experiment) in DMEM/F12+N2; Dox, Dox (12.5 ng/ml, equal to the levels of Dox in diluted hMSCs G/G + Dox medium used in the experiment) in DMEM/F12+N2

It is known that the withdrawal of serum from the cultures provokes a relatively rapid degeneration of DA neurons over a few days [41], [42]. We used this serum-free culture system to examine potential neuroprotective effects of hMSCs G/G +Dox conditioned medium on DA neurons. The results of TH immunocytochemistry showed that the treatment of hMSCs G/G +Dox conditioned medium improved the survival of DA neurons (Fig. 8A) when compared to the treatment of hMSCs G/G − Dox (Fig. 8B), hMSCs (Fig. 8C), Control (Fig. 8D) or Dox (Fig. 8F) conditioned medium. This neuroprotective effect of hMSCs G/G + Dox conditioned medium was found to be similar to that with the treatment of GDNF protein (Fig. 8E). Quantification data showed that cell body area, the number of neurites and the number of branching points for dendrites, and the proportion of TH-immunoreactive (TH-IR) cells in the cultures were all significantly greater in the treatment of either hMSCs G/G + Dox (248.68±20.89 µm^2^, 4.69±0.38, 4.15±0.52, and 182.58±9.85%) or GDNF (251.82±15.66 µm^2^, 4.64±0.33, and 4.91±0.51, and 169.34±8.62%) conditioned medium than those in the cultures incubated in the Control medium (194.25±9.63 µm^2^, 3.09±0.16, 2.09±0.25, and 100.02±8.54%) (Fig. 9A, B, C, and D) (one-factor ANOVA followed by Fisher’s post hoc test, F (5, 152)  = 7.25, F (5, 254)  = 23.09, F (5, 254)  = 13.19, F (5, 75)  = 19.80, * p<0.05). In contrast, the treatment of hMSC (180.63±5.56 µm^2^, 2.21±0.18, 1.21±0.28, and 89.25±11.18%), hMSCs G/G – Dox (189.12±10.46 µm^2^, 2.57±0.20, 1.36±0.31, and 119.07±6.48%), or Dox (177.94±9.64 µm^2^, 2.58±0.07, 2.14±0.12, and 100.39±7.25%) conditioned medium did not result in significant changes in the parameters of cell body area, the number of neurites and branching points for dendrites, and the proportion of TH-IR cells in the cultures when compared to those in the cultures incubated in the Control medium (p > 0.05).

## Discussion

In the present study, we generated a binary Tet-On lentivirus vector system based on two lentivirus vectors: a bicistronic vector expressing both hGDNF and hrGFP genes under a TRE-minimal CMV promoter, and a vector expressing rtTA2S-M2 under a minimal CMV promoter. We further characterized this system in hMSCs as examined inducibility of transgene expression, background expression, and function of transgene product, hGDNF. The results show that hMSCs transduced with inducible lentivirus vectors efficiently express both the hGDNF and hrGFP transgenes in the presence of Dox. The background expression of the two genes in transduced hMSCs is very low in the absence of Dox. Conditioned medium from cultures of transduced hMSCs in the presence of Dox protects SH-SY5Y cells from 6-OHDA-induced toxicity, and improves the survival of dopaminergic neurons from ventral mesencephalic (VM) tissue *in vitro*.

GDNF has proved to be a potent neurotrophic factor for DA neurons [1]–[4]. However, several studies have also demonstrated that continuous overexpression of GDNF mediated by virus vectors results in adverse side effects for DA system in the brain [8], [26], [27]. It has been shown that lentivirus-mediated long-lasting overexpression of the GDNF gene in the striatum results in aberrant sprouting and downregulation of TH activity in lesioned nigrostriatal dopamine neurons [26]. For the intact striatum, long-term over-expression of the GDNF gene can lead to downregulation of TH activity [8], [27]. In addition, Winkler et al. showed that continuous exposure to GDNF for nigral grafts in the striatum impaired graft-mediated function recovery [43]. A gene regulation system is therefore required to control GDNF levels for GDNF therapy. Although much effort has been made to improve inducible lentivirus vectors, lentivirus-mediated gene regulation is still unsatisfactory. By injecting inducible lentivirus vectors harboring the GDNF gene into the striatum, Georgievska et al., reported that GDNF gene expression was regulated with the tetracycline-regulated lentivirus vector system associated with a significant background leakage [38]. In an *ex vivo* gene delivery study, Behrstock et al. showed that transplantation of human neural progenitor cells transduced with Tet-Off lentivirus vectors carrying the GDNF gene did not lead to down-regulation of GDNF in response to doxycycline in the striatum of a rat model of PD [13]. Inability of gene regulation may represent a big obstacle for lentivirus vectors to be viable virus vectors in clinical application.

In the present study, we confirmed and extended the previous finding and showed that the modified binary Tet-On lentivirus vector system tightly regulated hGDNF and hrGFP transgene expression with a little leakage in the absence of Dox. The modifications to the binary Tet-On lentivirus vector system may attribute to the efficient inducibility of transgene expression and low background expression [29]. As the intact U3 region in the viral LTR of lentivirus vectors has promoter or enhancer elements which may attribute to background expression of transgenes, the inducible lentivirus vectors were made SIN by deleting 400 bp from the U3 region of the 3′ LTR [44]. The 1.2-kb chicken β-globin insulator element cHS4 was incorporate into the inducible lentivirus vectors at the site of the U3 deletion. Previous studies have demonstrated that cHS4 element reduces background expression in inducible adenovirus and adeno-associated virus vectors [45], [46]. In addition, the second-generation TREs (TRE/Pitt) were incorporated into the transgene expression vector. Agha-Mohammadi et al. showed that the minimal CMV promoter and tetO sequences were positioned in an optimized manner relative to the start of the transcript [39]. Indeed, by dose-dependent study, a very low dose of Dox (0.1 ng/ml) induced the hrGFP gene to express in both HeLa cells and hMSC transduced with binary Tet-On lentivirus vectors. The data from FCM, GDNF ELISA, and qRT-PCR confirmed that transduction of hMSCs with the binary Tet-On lentivirus vector system resulted in tightly regulation of hGDNF and hrGFP transgene expression. The expression of hGDNF and hrGFP transgenes in transduced hMSCs was efficiently switched “On” and “Off” by the addition or removal of Dox. With support to the previous study, our binary Tet-On lentivirus vector system induced hGDNF transgene expression in transduced hMSCs in the presence of Dox up to 719-fold above background levels as measured the levels of hGDNF protein in conditioned medium. In the “On – Off” experiment, we noticed that a certain period of time was required to wash out transgene expression after the removal (Off) of Dox. The changes in reduction of hGDNF transgene expression seemed to be slower than those in reduction of hrGFP transgene expression. The difference between GFP and GDNF gene regulation was also observed in a previous study in which a Tet-Off lentivirus vector system was used to transduce human neural progenitor cells [13]. In their system, gene regulation was very efficient for the marker gene, GFP expression, but GDNF transgene expression was only partially switched off in response to doxycycline *in vitro*. The properties of transgene products may contribute to this difference. GDNF has a long half-life and GFP is an unstable protein. In the present study, transduced hMSCs were able to be induced to differentiate into other cell types, adipogenic and osteogenic cells, suggesting that transduction of our binary Tet-On lentivirus vectors carrying both the hGDNF and hrGFP genes did not alter the cell properties of hMSCs. The current study showed that transduced hMSCs were stable over multiple passages, and consistently released the great levels of hGDNF into the medium. This makes it possible to bank and characterize the cells prior to transplantation. It is also worthy of note that the bicistronic lentivirus vector allows the simultaneous expression of the hGDNF and hrGFP genes. Expression of the marker gene, hrGFP is significant for basic research because it facilitates the purification of transduced cells to get a large quantity of the cells *in vitro* by cell sorting prior to transplantation, and tracing implanted cells in the brain.

The function of hGDNF in conditioned medium was examined in two cell culture model systems: SH-SY5Y cells exposed to 6-OHDA and nigral DA neurons in serum-free culture conditions. GDNF has been well documented to protect DA cells against 6-OHDA-induced toxicity, possibly through the phosphatidylinositol 3-kinase (PI3K) and mitogen-activated protein kinases (MAPK) signaling pathways [47]–[49]. The withdrawal of serum from the VM tissue cultures can provoke rapid degeneration of DA neurons [41], [42]. Under serum-free conditions, glia-mediated trophic support to DA neurons is virtually lost as the majority of glial cells are selectively removed from total surviving cells in cultures. In addition, DA neurons are believed to be dependent on the antioxidant glutathione system within glial cells. The loss of glial cells under serum-free conditions may accelerate the degeneration of DA neurons. Accumulation of free radicals in DA neurons leads to the gradual consumption of endogenous antioxidants, leading to an excess of reactive species to damage the cells. Numerous studies have shown that GDNF promotes the survival, cell density and differentiation of cultured VM neurons [50]–[52]. Sawada et al. showed that GDNF exerted neuroprotection against apoptosis through the PI3K signaling pathway and the subsequent up-regulation of Bcl-2 and Bcl-x [53]. Here we demonstrated that significant levels of hGDNF protein were detected in the hMSCs G/G + Dox conditioned medium. The treatment of the hMSCs G/G + Dox conditioned medium protected SH-SY5Y cells against 6-OHDA-induced cell loss as measured by MTT assay, and promoted the survival of cultured nigral DA neurons as examined by cell body area, number of neuritis and branching points, and proportion of TH-IR cells. The neuroprotection of the hMSCs G/G + Dox conditioned medium was similar to that in the GDNF control, suggesting that hGDNF in the hMSCs G/G + Dox conditioned medium plays a critical role in neuroprotection. The treatment of the hMSCs G/G – Dox conditioned medium failed to protect both SH-SY5Y cells and cultured nigral DA neurons, further confirming that the background expression of hGDNF gene was very low, if there was any. It is noted that hMSCs without virus transduction do not have neuroprotective effects. Although several studies have reported that hMSCs can produce a number of cytokines and growth factors [54], [55], we speculate that hMSCs-produced growth factors may not be sufficient enough to protect cells in our cell model system.

In summary, we developed an inducible cellular hGDNF gene delivery system which can tightly regulate hGDNF and hrGFP transgene expression with a little leakage of the system in the absence of Dox. hGDNF released from transduced hMSCs is functional although further studies are warranted to examine the mechanisms underlying the neuroprotection of hGDNF. It is imperative to examine if the system works *in vivo*. Our inducible cellular hGDNF gene delivery system may provide useful tools for basic research on gene therapy for chronic neurological disorders such as PD.

## Materials and Methods

### Plasmids and virus vector production

In a previous study, Pluta et al. developed an improved binary Tet-On system based on two lentivirus vectors, transfer vectors harboring the EGFP gene under second-generation TRE and rtTA2S-M2 vectors carrying a gene encoding advanced rtTA - rtTA2S-M2 under a human minimal CMV-IE promoter with efficient regulation of transgene expression [29]. In the present study, we modified this system and generated a binary Tet-On lentivirus vector system to deliver both the hGDNF and hrGFP genes (Fig. 1). Transfer plasmid, pNL-TRE/Pitt-hGDNF-IRES-hrGFP-ΔU3 was constructed by removing a *BamHI/BsrGI* fragment containing the EGFP sequence and replacing it with a *BamHI/XhoI* fragment containing hGDNF-coding sequence derived from pLVT-hGDNF-rtTRKRAB2SM2, provided by Dr. Aebischer (Lausanne, Switzerland) [34], and a *XhoI/SmaI* fragment containing an internal ribosome entry site (IRES)-hrGFP sequence derived from pIRES-hrGFP (Stratagene, USA), as shown in Figure 1. This bicistronic lentivirus vector allows the simultaneous expression of the hGDNF and hrGFP genes. The key element of these vectors is an optimized IRES which permits two genes of interest to be co-expresssed as separate proteins from a single mRNA transcript. rtTA2S-M2 plasmid, pNL-CMV-IE-rtTA2S-M2-ΔU3 was also prepared. Three plasmids, the transfer or rtTA2S-M2 plasmid, pCMVdR8.74 and pMD2VSV.G plasmids (provided by Dr. Didier Trono, Lausanne, Switzerland) were used for packaging lentivirus vectors. The lentivirus vectors were prepared by the transient transfection of 293T cells using the calcium phosphate precipitation method [40].

### Cell culturing and *in vitro* transduction

HeLa cells (American Type Culture Collection, ATCC, Manassas, VA, USA) were maintained in Dulbecco’s modified Eagle’s medium (DMEM) supplemented with 10% fetal bovine serum (FBS), and hMSCs [56] (kindly provided by Dr. Darwin J. Prockop at Center for Gene Therapy, Tulane University Health Sciences Center, New Orleans, USA) were maintained in DMEM supplemented with 16.5% FBS, 2 mM L-glutamine, and 1% penicillin/streptomycin at 37 °C under an atmosphere of 5% CO_2_ and 95% air. Tet-free FBS (Clontech, USA) was used in the experiments to reduce the background leak of transgene expression. We transduced HeLa cells and hMSCs with both pNL-TRE/Pitt-hGDNF-IRES-hrGFP-ΔU3 and pNL-CMV-IE-rtTA2S-M2-ΔU3 lentivirus vectors at a ratio of 1∶2. The concentration of pNL-TRE/Pitt-hGDNF-IRES-hrGFP-ΔU3 vectors was a multiplicity of infection (MOI) of 5 and pNL-CMV-IE-rtTA2S-M2-ΔU3 vectors 10. Virus particles were washed away with a replacement of new culture medium after 8 h transduction. To examine dose-dependent expression of hrGFP transgene in HeLa cells and hMSCs, Dox in serial concentrations (ranging from 10^−4^ to 10^4^ ng/ml) was added to cultures 8 h post-transduction. hrGFP expressing cells were examined 4 days after Dox treatment using FCM. To examine the efficiency of regulated transgene expression by a binary Tet-On lentivrus vectors, “On-Off” and “Off-On” treatment groups were designed. In the “On-Off” group, transduced hMSCs were first incubated in the presence (On) of Dox (100 ng/ml) for 4 days, and then in the absence (Off) of Dox with a replacement of culture medium for additional 3, 6 and 10 days. In the “Off-On” treatment group, transduced hMSCs were first incubated in the absence (Off) of Dox for 4 days, and then in the presence (On) of Dox with a replacement of culture medium for additional 3, 6 and 10 days. At desired time points (0, 4, 7, 10, and 14 days after the first treatment with or without Dox), transduced hMSCs were prepared for FCM analysis to determine the MFI units of hrGFP. Transduced hMSCs were also prepared for qRT-PCR to determine mRNA copy numbers for hGDNF and hrGFP at 4 days. In addition, conditioned medium was collected from transduced hMSCs in the presence or absence of Dox for hGDNF ELISA (Promega, USA) to determine protein levels of hGDNF in culture medium at 0, 4, 7, 10, and 14 days. After collecting culture medium at each time point, fresh culture medium was added to cultures.

### FCM analysis

Transduced HeLa cells and hMSCs in cultures were digested with 0.25% trypsin/EDTA (Sigma, USA) and centrifuged at 1000 rpm for 5 min, the supernatant was then removed. The cells were washed twice with phosphate buffered saline (PBS) and adjusted to a concentration of 1×10^6^ cells/ml for FCM analysis to determine the MFI units for hrGFP^+^ or hrGFP^+^ cell counts.

### qRT-PCR

The copy number of hGDNF mRNA was determined by qRT-PCR with the RNA samples isolated from transduced hMSCs. Total RNA was extracted using the Qiagen Rneasy kit (P/N74104), then treated twice with DNase TURBO DNA-free (Ambion, Austin, TX). 2 µg of total RNA was converted to cDNA with SuperScript II reverse transcriptase (Invitrogen) and an oligo-dT (12–18) primer (Invitrogen). TaqMan PCR was conducted with an ABI PRISM 7700 system (Applied Biosystems, Foster City, CA) in a total volume of 50 µl. The PCR mix contained 25 µl of 2×TaqMan Universal PCR Master Mix (Applied Biosystems), 10 pmol each of the forward and reverse primers and probe. The sequences of primers and probe specific for hGDNF were as follows: forward primer, 5′–CTGACTTGGGTCTGGGCTATG-3′; reverse primer 5′–TTGTCACTCACCAGCCTTCTATTT-3′; probe, 5′-TGCGATGCAGCT GAGACAACGTACG-3′. The sequences of primers and probe specific for hrGFP were as follows: forward primer, 5′– ACCTGATCGAGGAGATGTTCGT-3′; reverse primer 5′– AGGCCGGTGATGGTCTTCTT-3′; probe, 5′- CAAGGGCCGCAACTTCCCCAAC-3′. Human β-actin was used as an internal control to normalize the results of hGDNF and hrGFP mRNAs in each sample (forward primer: 5′–GCGAGAAGATGACCCAGATC-3′, reverse primer: 5′–CCAGTGGTACGGCCA GAGG-3′, probe: 5′-CCAGCCATGTACGTTGCTATCCA GGC-3′). Serial dilutions of plasmid DNA of transfer plasmid, pNL-TRE/Pitt-hGDNF-IRES-hrGFP-ΔU3 were used to establish standard curves. The cycling conditions were 2 min at 50°C, and 10 min at 95°C, then 40 cycles of 15 sec at 95°C and 1 min at 60°C. The data were analyzed using Sequence Detector software version 1.7 (Applied Biosystems).

### *In vitro* differentiation assay

To examine if transduction of hMSCs with binary Tet-On lentivirus vectors altered stem cell properties of hMSCs, differentiation ability of hMSCs was examined. Transduced hMSCs were plated at 1×10^5^ per well into three wells per group in a six-well plate. The cells in two wells were induced to differentiate into either osteogenic or addipogenic cells using specific differentiation medium. The cells in one well remained undifferentiated as a control. The cells were incubated in complete culture medium until 100% confluence, then medium was changed to either osteogenic (10^−8^ M dexamethasone, 0.2 mM ascorbic acid, 10 mM β-glycerolphosphate; Sigma) or adipogenic medium (0.5 µM hydrocortisone, 0.5 mM isobutylmethylxanthine, 60 µM indomethacin). The cells were maintained for 3 weeks *in vitro* with a change of culture medium every 4 days. The cells were fixed with 4% paraformaldehyde and stained with Alizarin Red (Sigma) for osteogenic cells or Oil Red O (Sigma) for adipogenic cells.

### Collection of conditioned medium

Transduced hMSCs were maintained in DMEM supplemented with 16.5% FBS, 2 mM L-glutamine, 100 units/ml penicillin/streptomycin, at a density of 2×10^4^ cells/mL in 75 cm^2^ tissue flasks, at 37°C under an atmosphere of 5% CO_2_ and 95% air for 3 days. Then medium was replaced with serum-free DMEM/F12 in the presence or absence of Dox (100 ng/ml). At 7 day, conditioned medium from the cultures in the presence (denoted as hMSCs G/G + Dox) or absence (hMSCs G/G – Dox) of Dox were collected and stored at –80°C for the following experiments. Before collection of conditioned medium, transduced hMSCs were also examined and photographed using an inverted fluorescence microscope, and prepared for FCM analysis to determine the percentage of hrGFP^+^ cells. In addition, conditioned media from cultures of hMSCs without lentivirus transduction (hMSCs) were also collected.

### hGDNF ELISA

The protein levels of hGDNF in conditioned media (hMSCs G/G – Dox, hMSCs G/G + Dox, and hMSCs) were determined using hGDNF ELISA (Promega, USA) according to the manufacturer’s instructions. Briefly, a ninety-six well ELISA plate was coated with 100 µl of buffer containing 0.1 µl anti-GDNF monoclonal antibody for overnight at 4°C. The wells were then blocked with 200 µl of 1 × block & sample buffer for 1 hour at room temperature. 100 µl of diluted GDNF standard protein and conditioned medium (1∶80, 1∶160, 1∶320 of hMSCs G/G + Dox group, and no dilution of the other groups) were added to wells with shaking for 6 hours at room temperature. The wells were washed and incubated with 100 µl of anti-GDNF polyclonal antibody (1∶500) overnight at 4°C. The wells were washed and incubated with 100 µl of HRP-conjugated anti-chicken IgY for 2 hour at room temperature, then washed and developed with TMB one solution. The wells were loaded 100 µl of 1 N hydrogen chloride to stop development and read at 450 nm with a microplate reader. The levels of hGDNF protein were presented as the optical density (OD) value.

### SH-SY5Y cell culture and 6-hydroxydopamine (6-OHDA) treatment

SH-SY5Y cells were maintained in DMEM supplemented with 10% FBS at 37 °C under an atmosphere of 5% CO_2_ and 95% air. Culture medium was changed twice a week. To insult SH-SY5Y cells, stock solution of 6-OHDA (5 mM) were prepared in cold saline containing 0.15% ascorbic acid and used for all experiments. To determine an appropriate dose of 6-OHDA, we first performed dose-dependent cell viability assays. SH-SY5Y cells were treated with 6-OHDA at different concentrations (ranging from 20 µM to160 µM) for 16 hours. To examine effects of hMSCs G/G + Dox conditioned medium on cell viability of SH-SY5Y cells, the cells were treated with hMSCs G/G + Dox conditioned medium with serial dilutions (1∶1, 1∶2, 1∶4, 1∶8, 1∶16, 1∶32, 1∶64, and 1∶128) for 16 hours. To examine which concentrations of hMSCs G/G + Dox conditioned medium protected SH-SY5Y cells against 6-OHDA-induced toxicity, SH-SY5Y cells were co-incubated with 6-OHDA (100 µM) and hMSCs G/G + Dox conditioned medium at serial concentrations for 16 hours. To examine specificity of protective effects for hMSCs G/G + Dox conditioned medium, SH-SY5Y cells were co-treated with 6-OHDA (100 µM) and various conditioned media (hMSCs, hMSCs G/G – Dox, hMSCs G/G + Dox) for 16 hours. In addition, DMEM/F12 containing GDNF (GDNF, 11.68 ng/ml, equal to the levels of GDNF in diluted hMSCs G/G + Dox medium, 1∶8, used in the experiment), DMEM/F12 containing Dox (Dox, 12.50 ng/ml, equal to the levels of Dox in diluted hMSCs G/G + Dox medium used in the experiment) and DMEM/F12 were also used as controls. Cells viability of SH-SY5Y cells was determined by the conventional 3, [4,5-dimethylthiazol-2-yl]- diphenyltetrazolium bromide (MTT) assay (Sigma, USA).

### Determination of cell viability

SH-SY5Y cells viability was determined by MTT using a MTT assay kit according to the manufacturer’s instructions. Briefly, SH-SY5Y cells were plated at a density of 1.0 × 10^4^ cells/ well in a 96-well plate, pre-coated with poly-D-lysine (Trevigen, USA). Cells were treated with the MTT solution (final concentration, 0.5 mg/ml) for 4 hours at 37°C. The medium was replaced with 100 µl of DMSO for each well. The formazan dye crystals were solubilized for 30 min, and absorbance at 570 nm was measured with a microplate reader (Molecular Devices, Sunnyvale, CA, USA). Results were expressed as the percentage of MTT reduction, assuming that the absorbance of control cells was 100%.

### Preparation of ventral mesencephalic tissue cultures

All animal procedures were carried out in strict accordance with the recommendations in the Guide for the Care and Use of Laboratory Animals of the National Institutes of Health, and approved by the Animal Use and Care Committee of Capital Medical University, Beijing, China. The number of animals used was the minimum required for statistical analysis, and all precautions were taken to minimize animal suffering. All surgery was performed under sodium pentobarbital anesthesia (100 mg/kg body weight, i.p.), and animals were sacrificed with an overdose of sodium pentobarbitone (200 mg/kg body weight, i.p.) in the end of experiments. Pregnant Sprague-Dawley (SD) rats at gestation day 14 were obtained (Vital River Laboratory Animal Technology, China). VM tissue was dissected from the brain of embryos on ice and trypsinized into single-cell suspension with 0.25% trypsin/EDTA for 10 minutes at 37°C. The cells were re-suspended in DMEM using sterilized micropipette tips, supplemented with 10% FBS and plated at a final density of 2×10^5^ viable cells/well in four-well chamber slides (Thermo Scientific Nunc Lab-Tek II Chamber Slide, USA). The chamber slides were pre-coated with 500 µl of poly-D-lysine (20 µg/ml) for 2 hours at 37°C. The single cells were incubated at 37°C under atmosphere of 5% CO_2_ and 95% air for 2 days. To examine the functional effects of hGDNF released by transduced hMSCs in the presence of Dox, the medium was switched to new serum-free DMEM/F12 supplemented with N_2_ medium. At this time point, hMSCs G/G + Dox, hMSCs G/G – Dox, hMSCs, GDNF, Dox, or DMEM/F12+N2 conditionedmedium was added to VM tissue cultures for additional 7 days *in vitro*.

### Immunocytochemistry

VM tissue cultures were fixed at day 7 by 4% paraformaldehyde in PBS for 20 minutes at room temperature. The avidin-biotin complex immunoperoxidase technique was used to visualize TH immunoreactivity. The primary antibody used was against TH (rabbit polyclonal antibody, 1∶300, Santa Cruz Biotechnology). The secondary antibody was biotinylated goat anti-rabbit (1∶200, Vector Laboratories, Burlingame, CA, USA). Cells were incubated in ABC solution (Vectastain ABC Elite kit, Vector Laboratories, USA) followed by development with 3,3′-diaminobenzidine solution (Vectastain DAB kit, Vector Laboratories, USA) to visualize immunoreactivity. To evaluate the specificity of immunostaining, normal goat serum was used instead of a primary antibody, or primary antibody was omitted during the immunostaining as a negative control.

### Morphological assessment

TH-immunostained cell cultures were examined at 200× and 400× magnification using light microscope (Nikon, Japan) with bright field illumination in a blinded manner. The original codes of the slides, which indicated treatment groups, were covered by opaque tape and the slides were re-numbered. After evaluation, the original codes were revealed. TH-IR cells in cultures were examined in a 400 µm^2^ ocular grid which was randomly placed at 4 predetermined sites of each well. TH-IR cells were only evaluated in an ocular grid which covers a field containing at least 4 TH-IR cells. TH-IR cells in about 36 fields were examined for each treatment group. By 200× magnification, the number of neurites and branching points for dendrites were counted, and cell body area of TH-IR cells were quantified using HCImage software (HCImage, 3.0, Hamamatsu, Japan). By 400× magnification, the number of TH-IR cells and total cells in a field was counted, and the proportion of TH-IR cells in the total cells was calculated and presented as a percentage of the control without conditioned medium.

### Statistical analysis

For all *in vitro* experiments, at least three replicas per group were used in each experiment. Data were collected from at least three independent experiments, and were presented as the mean ± standard error of the mean (SEM). All data were subjected to statistical analysis using StatView software. A one-factor ANOVA followed by Fisher’s post-hoc test was used for group comparisons. Statistical significance was defined at P<0.05.
